# Supplementary material for: Using drones to improve care for HIV-exposed children in Conakry, Republic of Guinea: Anthropological perspectives
Source: PLOS Glob Public Health. 2024 Nov 27;4(11):e0003445. doi: 10.1371/journal.pgph.0003445 (PMC11602074; doi:10.1371/journal.pgph.0003445)
Supplement: Attachment — Submitted filename: Response_Drone_R1_30062024.docx [file pgph.0003445.s003.docx]

**RESPONSE TO EDITORS COMMENTS**

PGPH-D-23-02263R1

Using drones to improve care for HIV-exposed children in Conakry, Republic of Guinea: anthropological perspectives

==============================

Thank you for submitting your manuscript entitled "Using drones to improve care for HIV-exposed children in Conakry, Republic of Guinea: anthropological perspectives" to PLOS Global Public Health. Your manuscript files have been checked in-house, but before we can proceed we need you to address the following issues:

1. Please amend your detailed online Financial Disclosure statement. This is published with the article. It must therefore be completed in full sentences and contain the exact wording you wish to be published.
a) State the initials, alongside each funding source, of each author to receive each grant. For example: "This work was supported by the National Institutes of Health (####### to AM; ###### to CJ) and the National Science Foundation (###### to AM)."
b) State what role the funders took in the study. If the funders had no role in your study, please state: “The funders had no role in study design, data collection and analysis, decision to publish, or preparation of the manuscript.”

**Response:** **Financial Disclosure statement have been completed, we have added the initial of the PI author who received the grant. We have also indicated the role of the funder.**

2. Please update your online Competing Interests statement. If you have no competing interests to declare, please simply state: “The authors have declared that no competing interests exist.”

**Response: done**

3. We note your current Data Availability statement is:

"The data is sensitive because it is potentially identifying. Excerpts from anonymized interviews are included in the article. Data is available upon request to the authors."
PLOS journals require authors to make all data necessary to replicate their study’s findings publicly available without restriction at the time of publication. When specific legal or ethical restrictions prohibit public sharing of a data set, authors must indicate how others may obtain access to the data.
Furthermore, PLOS’ Data Policy states that authors cannot be the sole named individuals responsible for ensuring data access (https://journals.plos.org/plosone/s/data-availability#loc-unacceptable-data-access-restrictions).
Therefore, before we can proceed, please also provide non-author contact information for a data access committee, ethics committee, or other institutional body to which data requests may be sent.
We will update your Data Availability statement on your behalf to reflect the information you provide.

**Response: We have had a series of exchanges with Dr Rashmi Josephine Rodrigues (4/22/2024) and then with Dr Jacob Ulmschneider (4/24/2024). We confirm that pseudonymized transcripts are our primary data set. Pseudonyms were used in the transcription of interviews, but given the precision of the information collected on individuals, it is impossible to completely anonymize the data, which remains potentially identifying.**

**Following the recommendations of Dr Jacob Ulmschneider, we have drawn up a new Data Availability statement that includes the reason for the restrictions. We have specified that metadata relating to the article is available in an open data repository and access to limited, anonymous information contained in the interview transcripts can be obtained on reasoned request by e-mail to:** [**data@ird.fr**](mailto:data@ird.fr)**, which is a non-author contact information for a data access.**

**So, the new Data Availability statement is : The data is sensitive because it is potentially identifying. In compliance with the confidentiality obligations recommended by the National Health Research Ethics Committee of Guinea, mentioned in the consent form, it is not possible to share the full transcript of the interviews. The metadata related to this article (protocol, interview guide, book code, main socio-demographic characteristics of interviewees) are accessible on the DataSud repository (**[**https://doi.org/10.23708/L1KYFP**](https://doi.org/10.23708/L1KYFP)**). Access to limited, anonymous information contained in the interview transcripts can be obtained on reasoned request by e-mail to:** [**data@ird.fr**](data@ird.fr)

4. We do not publish any copyright or trademark symbols that usually accompany proprietary names, eg (R), (C), or TM (e.g. next to drug or reagent names). Please remove all instances of trademark/copyright symbols throughout the text, including ® on pages 6 and 10.

**Response: we have removed the trademark symbols.**

***
