# Supplementary material for: Using drones to improve care for HIV-exposed children in Conakry, Republic of Guinea: Anthropological perspectives
Source: PLOS Glob Public Health. 2024 Nov 27;4(11):e0003445. doi: 10.1371/journal.pgph.0003445 (PMC11602074; doi:10.1371/journal.pgph.0003445)
Supplement: Attachment — Submitted filename: Response to editors comments_R1_280824.docx [file pgph.0003445.s004.docx]

**RESPONSE TO EDITORS COMMENTS**

PGPH-D-23-02263R1
Using drones to improve care for HIV-exposed children in Conakry, Republic of Guinea: anthropological perspectives
PLOS Global Public Health
==============================

Journal Requirements:

1. Please review your reference list to ensure that it is complete and correct. If you have cited papers that have been retracted, please include the rationale for doing so in the manuscript text, or remove these references and replace them with relevant current references. Any changes to the reference list should be mentioned in the rebuttal letter that accompanies your revised manuscript. If you need to cite a retracted article, indicate the article’s retracted status in the References list and also include a citation and full reference for the retraction notice.

**Response: We have carefully reviewed the list of bibliographical references. We have not cited any articles that have been retracted.**

6. Review Comments to the Author

Reviewer #1: I commend the authors for thoroughly reviewing the initially submitted manuscript draft. They have sufficiently addressed the comments raised in the initial review and presented a higher quality draft considering that English may not be their primary language which could have affected the initial expression of thoughts.

**Response: thank you for your appreciation**

Generally, the authors should consider reviewing the results section for consistency of tenses used. Currently the authors use present continuous tense and past tenses interchangeably. Since they are reporting past findings, the past tense is recommended for reporting the results.
**Response: Done : we have taken your comments into account and revised the text accordingly.**

New comment:

The authors could consider highlighting the strengths, if any, of their study, not just the limitations.
**Response: Following your advice, we have added the paragraph below specifying the strengths of our research.**

**“Thus, despite the limitations of the study mentioned above, the strengths of our study lie in our attempt to gather the views of the various social actors who are likely to be affected by the use of drones. This research is part of the anthropological contribution to the analysis and understanding of the acceptability of health innovations. From a public health perspective, our analyses provide elements for optimizing health programs using drones and to maximise public acceptance. Our findings deserve to be shared and compared with those of other countries, to take account of the factors influencing the acceptability of the use of drones in healthcare programmes, in a context where such programmes are growing rapidly in Africa.”**

Line by line comments:

Line 106: Remove ‘of’ in United Nations International Children's Emergency Fund

**Response: done**

Line 244: Has a typographical error, “lassting”

**Response: done**

Line 373: Add ‘to’ HIV-positive children

**Response: done**

Line 512: “Raised by drones” sound like drones have raised the issue. Consider, “raised about drones”
**Response: done**

Reviewer #2: Authors addressed all comments as desired. The revised article looks OK to me.

***
